# Lipid-induced hepatic insulin resistance

**DOI:** 10.18632/aging.100585

**Published:** 2013-08-05

**Authors:** Thomas Galbo, Gerald I. Shulman

**Affiliations:** ^1^ Department of Internal Medicine, Yale University School of Medicine, New Haven, CT 06510, USA; ^2^ Department of Cellular and Molecular Physiology, Yale University School of Medicine, New Haven, CT 06510, USA; ^3^ Howard Hughes Medical Institute, Yale University School of Medicine, New Haven, CT 06510, USA; ^4^ Novo Nordisk Center for Basic Metabolic Research Copenhagen, Denmark

The principal function of insulin in the liver is to suppress glucose production when blood glucose concentrations increase. This process is impaired in hepatic insulin resistance and contributes to postprandial hyperglycemia. The development of hepatic insulin resistance is very closely linked to non alcoholic fatty liver disease (NAFLD), and is a major factor in the pathogenesis of type 2 diabetes (T2D). Yet, the molecular mechanisms regarding the pathogenesis have remained elusive.

**Figure 1:**
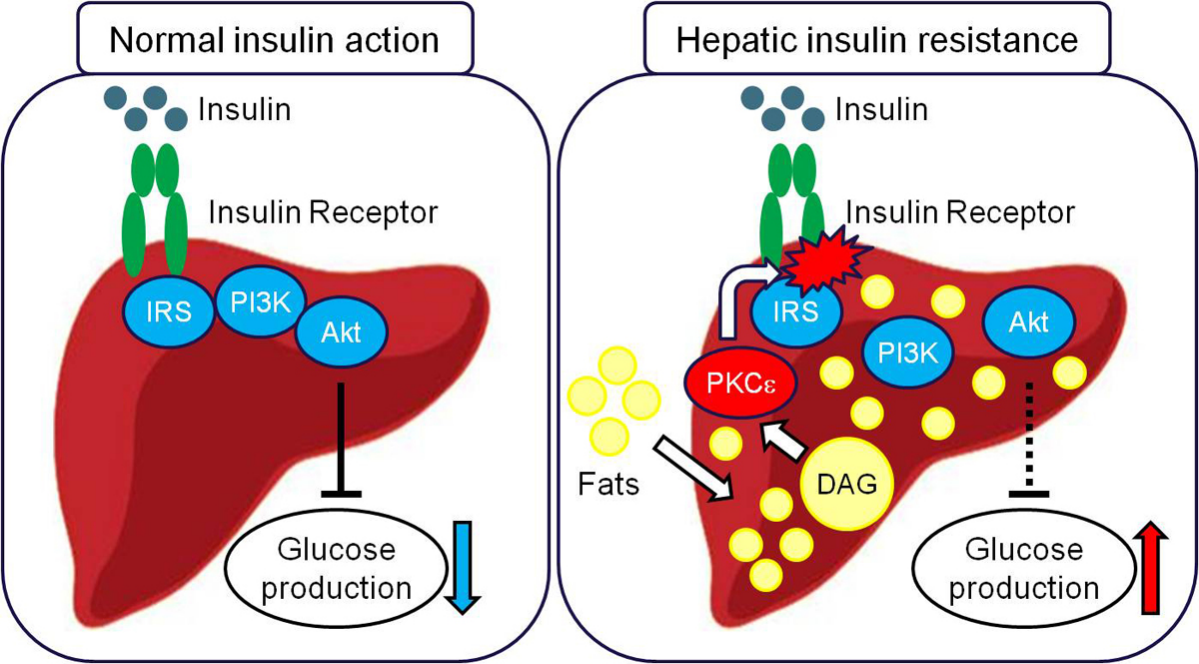
In normal insulin action (left panel), insulin binds and activates the insulin receptor kinase leading to phosphorylation of IRS proteins, recruitment and activation of PI3K, activation of Akt and ultimately suppression of glucose production and stimulation of glucose uptake and storage as glycogen. Recent studies, have indicated that in hepatic insulin resistance this signaling mechanism is defective (right panel). Hepatic accumulation of diacylglycerols (DAGs) due to: 1) increased delivery of fatty acids to the liver, 2) increased *de novo* lipogenesis and/or 3) decreased hepatic fat oxidation triggers PKCε activation which in turn inhibits insulin receptor kinase activity leading to disruption of downstream insulin signaling, thus resulting in an inadequate ability of insulin to suppress hepatic glucose production and glucose uptake and storage as glycogen.

Among several proposed mechanisms, two main schools of thought have gained support for hepatic insulin resistance associated with NAFLD. Briefly, the first proposes that excess lipid delivery to the liver and/or reductions in fatty acid oxidation, results in accumulation of intracellular diacyglycerols (DAGs). This increase in hepatic DAG content leads to activation of PKCε which, in turn, inhibits insulin-stimulated insulin receptor kinase phosphorylation of IRS proteins and impairs activation of downstream signaling [1, 2] (Figure 1). According to the other school of thought, saturated fatty acids induce insulin resistance by activating inflammatory TLR-4 signaling through the adaptor protein MyD88 leading to increased *de novo* ceramide synthesis, accumulation of ceramides and ceramide-mediated inhibition of insulin signaling through inhibition of Akt phosphorylation [3]. In this model, TLR-4 signaling and ceramide synthesis are both critical for saturated fat-induced hepatic insulin resistance while unsaturated lipid-induced insulin resistance is independent of the TLR-4 receptor and ceramide synthesis [3].

In a recently published study from our group [4], we tested these hypotheses. The specific aims were to examine: 1) how saturated and unsaturated fat-feeding affects hepatic accumulation of DAGs and ceramides as well as hepatic insulin signaling, and 2) the postulated role of the TLR-4/MyD88 signaling pathway in mediating saturated fat-induced hepatic accumulation of ceramides and hepatic insulin resistance.

To address these aims, we conducted experiments in rats fed high fat diets enriched with either saturated or unsaturated fat for three days. This is a well-established rodent model of selective lipid-induced hepatic insulin resistance. Further, to evaluate the putative role of TLR-4 receptor signaling in saturated lipid-induced insulin resistance, we conducted studies in normal mice treated with antisense oligonucleotides (ASOs) knocking down hepatic TLR-4 or MyD88 expression as well as in TLR-4 deficient mice, and tested how hepatic insulin sensitivity and ceramide/DAG content was affected by exposure to saturated fats.

We found that saturated or unsaturated fat-feeding in rats resulted in steatosis, increased intrahepatic DAG content, PKCε activation and impairment of insulin-stimulated IRS2-associated PI3-kinase signaling. However, neither the saturated or unsaturated fat diet led to an increase in hepatic ceramide content thus dissociating ceramide content from hepatic insulin resistance in this model.

In further experiments we found that mice treated with ASOs, to knock down hepatic expression of TLR-4 or MyD88, and fed a high saturated fat diet were protected from developing hepatic steatosis, which could be attributed to decreased caloric intake potentially due to increased plasma TNFα concentrations. However, knockdown of TLR-4 or MyD88 did not protect these mice from saturated fat-induced hepatic PKCε activation or impairment of hepatic insulin signaling following a lard (saturated fat) gavage. The observations that knockdown of TLR-4 and MyD88 can alter hepatic lipid content by reducing food intake underline the critically important fact that genetic manipulations in immune pathways in rodents can often lead to subtle alterations in appetite, activity, energy expenditure or other parameters that affect development of ectopic lipid deposition and insulin resistance, ultimately leading to flawed interpretations of experimental data if not taken properly into account. We further found that mice deficient of the TLR-4 receptor were not protected from saturated fat-induced hepatic steatosis, hepatic DAG accumulation, PKCε activation or insulin resistance as assessed by hyperinsulinemic-euglycemic clamp studies, when the decreased caloric intake was compensated for. In contrast to the proposed mechanism where activation of TLR-4 leads to activation of hepatic ceramide synthesis [3], TLR-4 deficient mice had increased hepatic ceramide content when fed the saturated fat diet.

In conclusion we found that: 1) both saturated- and unsaturated fat-feeding results in hepatic DAG accumulation, activation of PKCε and hepatic insulin resistance through impairment of insulin signaling at the level of IRS-2, 2) both saturated and unsaturated fat feeding induced hepatic insulin resistance independent of changes in hepatic ceramide content in normal rodents, and 3) TLR-4 receptor signaling is not required for saturated fat-induced accumulation of ceramides, triglycerides, DAGs or impairment of hepatic insulin signaling and lipid-induced hepatic insulin resistance. Importantly these results translate to humans where recent studies have found that increased hepatic DAG content [5,6] and PKCε activation [5], and not ceramide content, are strongly associated with hepatic insulin resistance. Consistent with the critical role for hepatic lipid in the pathogenesis of hepatic insulin resistance and T2D, recent studies have found that caloric restriction rapidly lowers hepatic fat content and improves insulin sensitivity in T2D patients [7,8]. The combined implications of these studies are that excess fat (DAGs) in the liver, regardless of whether saturated or unsaturated, causes insulin resistance. Therefore future therapeutic strategies targeting hepatic insulin resistance should be aimed at lowering hepatic fat content and DAG-PKCε-signaling, and not at inhibiting inflammatory (including TLR-4) signaling or ceramide synthesis, to most effectively treat the root cause of T2D.
